# Bronchiolo-arterial fistula management in a patient with Loeys-Dietz syndrome using a multidisciplinary approach

**DOI:** 10.1093/jscr/rjag006

**Published:** 2026-01-27

**Authors:** Meredith Otley, Muhanned Kheder, Jeremy Wood, Chris Lightfoot, Michael Rivers-Bowerman, Mathieu Castonguay, Daniel French

**Affiliations:** Faculty of Medicine, Dalhousie University, 1276 South Park Street, Halifax, NS B3H 4R2, Canada; Department of Cardiac Surgery, Mazankowski Alberta Heart Institute, University of Alberta, 11220 83 Ave NW, Edmonton, AB T6G 2R3, Canada; Division of Cardiac Surgery, Department of Surgery, Dalhousie University, Queen Elizabeth II Hospital, 1276 South Park Street, Halifax, NS B3H 2Y9, Canada; Division of Interventional Radiology, Department of Radiology, Dalhousie University, Queen Elizabeth II Hospital, 1276 South Park Street, Halifax, NS B3H 2Y9, Canada; Division of Interventional Radiology, Department of Radiology, Dalhousie University, Queen Elizabeth II Hospital, 1276 South Park Street, Halifax, NS B3H 2Y9, Canada; Department of Pathology, Dalhousie University, Queen Elizabeth II Hospital, 1276 South Park Street, Halifax, NS B3H 2Y9, Canada; Division of Thoracic Surgery, Department of Surgery, Dalhousie University, Queen Elizabeth II Hospital, 1276 South Park Street, Halifax, NS B3H 2Y9, Canada

**Keywords:** hemoptysis, bronchiolo-arterial fistula, Loeys-Dietz syndrome

## Abstract

Bronchiolo-arterial fistulae are rare occurrences that can lead to hemoptysis. In general, persistent hemoptysis has a broad range of causes and management options. Surgery is often used in cases where conservative approaches have failed. In this case study, we present a 43-year-old woman with Loeys-Dietz syndrome, a connective tissue disorder, who developed chronic persistent hemoptysis after multiple aortic operations. The hemoptysis did not resolve with antibiotics, corticosteroids, and coil embolization. Based on multidisciplinary consensus, a left upper lobectomy was performed, with no recurrence of hemoptysis after 2 years of follow-up. Pathology revealed a bronchiolo-arterial fistula.

## Introduction

Historically, surgery was the mainstay of treatment for large-volume hemoptysis, but management with bronchoscopy and interventional radiology (IR) procedures is increasingly used as a first-line treatment [1]. However, a subset of patients still requires surgery, often in patients who are refractory to other treatments [2, 3]. This case report presents a patient with Loeys-Dietz syndrome (LDS), a genetic connective tissue disorder, who developed persistent hemoptysis refractory to conservative management that resolved after a lobectomy. Pathology revealed a bronchiolo-arterial fistula.

## Case

A 43-year-old female with LDS initially presented in 2018 with a type A dissection extending from the aortic root to the iliacs and into the right subclavian artery that was treated with supracoronary replacement of her ascending aorta. In July 2021, she underwent open repair of a descending thoracic aneurysm. In December 2021, there was a perforation of her aortic graft adjacent to a partially resected rib with corresponding thoracic pseudoaneurysm formation treated with thoracic aortic endograft repair. Later that month, she underwent a mechanical Bentall procedure for repair of a ruptured aortic arch, innominate artery aneurysm, left subclavian artery dissection, and left carotid dissection due to a leaking thoracic aortic aneurysm. Postoperatively, she developed a left hemothorax and underwent bronchoscopy, decortication, and rib resection.

**Figure 1:**
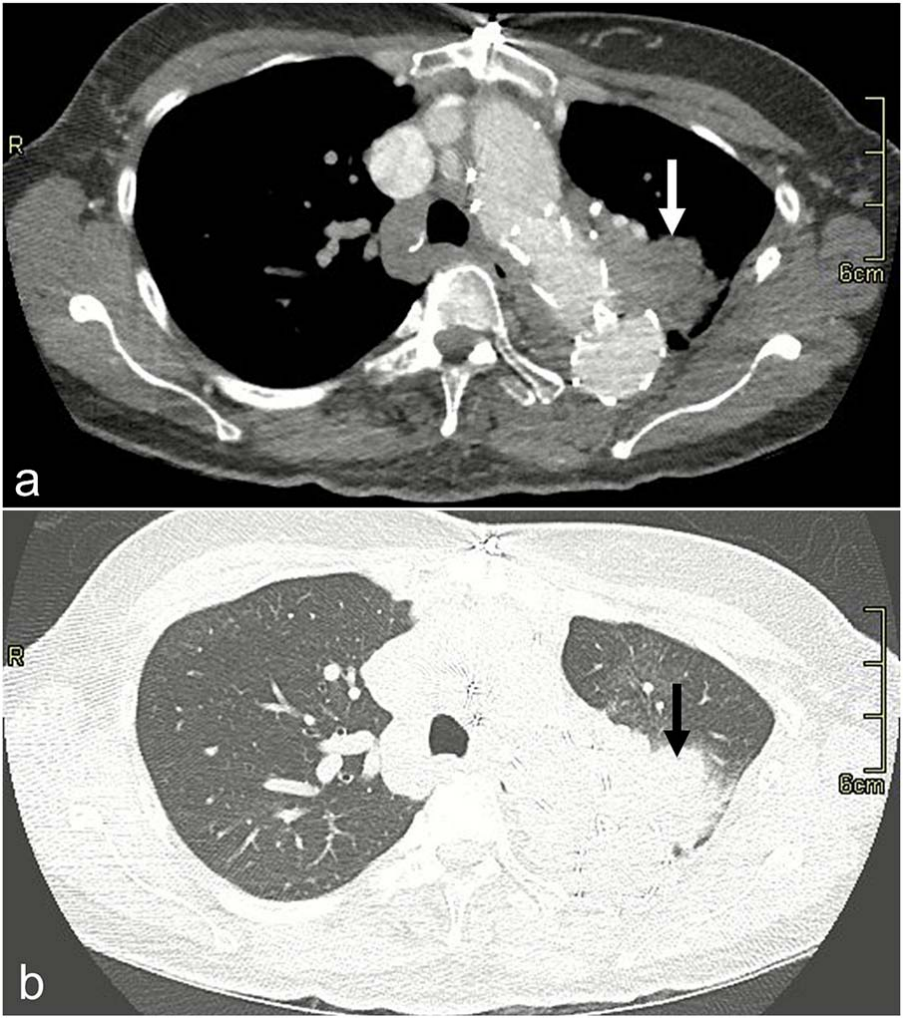
CT scan image with dissection protocol of the left upper lobe consolidation (arrow) on (a) mediastinal and (b) lung windows.

In 2022, she was admitted for recurrent hemoptysis. Bronchoscopy revealed a small amount of blood and inflammation in the left upper lobe (LUL), but no infectious source was identified on culture. Consolidation was identified on computed tomography (CT) in the LUL, which was suspicious for erosion of her graft resulting in pulmonary hemorrhage (Fig. 1). However, her hemoptysis decreased on antibiotics. The possibility of a fistula from her aortic graft to the lung parenchyma was raised, but an infectious process was favored due to her antibiotic response.

The hemoptysis recurred with periods of relief, and she underwent several courses of antibiotics and a course of corticosteroids. Her symptoms eventually escalated to daily hemoptysis with associated dyspnea. The consensus at a multidisciplinary case rounds with IR, cardiac, and thoracic surgery was that she should undergo an angiogram with possible embolization, with a lobectomy to be performed if this was unsuccessful.

**Figure 2:**
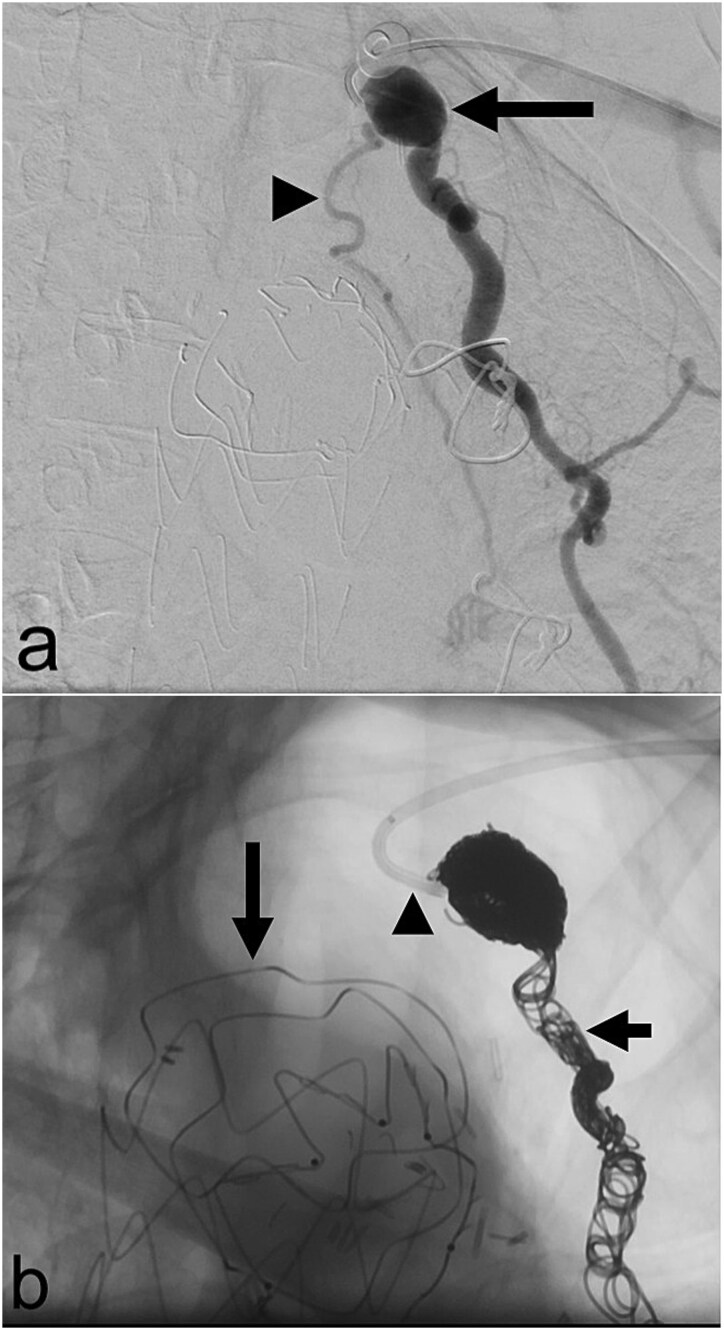
(a) Aneurysmal left internal mammary artery (arrow) with bronchial collateral (arrowhead) on digital subtraction angiography; (b) selective catheterization of the origin of the left internal mammary artery (arrowhead) via a left radial arterial approach with coil embolization of the left internal mammary artery (short arrow) and a thoracic aortic endograft (long arrow) on fluoroscopy.

In May 2023, she underwent coil embolization of an aneurysmal proximal left internal mammary artery (LIMA) giving rise to a bronchial collateral that was felt to be feeding the LUL lesion (Fig. 2). The collateral could not be subselectively catheterized. There was short-term resolution of her symptoms after which they recurred.

In August 2023, she was admitted for large volume hemoptysis associated with dyspnea. She was treated with corticosteroids and antibiotics decreasing but not resolving the hemoptysis.

Based on the patient’s clinical course, in September 2023, she underwent an open LUL lobectomy as a combined case with IR, cardiac, and thoracic surgery. Bilateral femoral arterial sheaths were placed to ensure adequate access in the event of rupture of the reconstructed aorta. A left thoracotomy was performed. Consistent with the discussion at the multidisciplinary case rounds, a decision was made early in the operation to perform a lobectomy rather than a lesser resection because of an inability to confidently identify the fistulous lesion and vascular structures due to dense adhesions. Decortication of the left hemithorax was required to release dense adhesions between the lung, pericardium, chest wall, diaphragm, and descending aorta. Accepting the risk of venous congestion, the left superior pulmonary vein was divided to facilitate exposure of the truncus branch of the artery and bronchus. The LUL pulmonary artery branches were divided after opening the fissure with cautery and staplers. The native aorta was adherent to the main pulmonary artery requiring careful dissection to separate these structures. Next the LUL bronchus was divided leaving only the LUL parenchyma attached to the aortic arch. To facilitate exposure, the majority of the LUL was amputated with serial firing of staplers leaving only a small remnant of LUL. This exposure revealed two large bronchial arteries under the arch of the aorta entering the LUL parenchyma. Two clips were applied to each vessel on the stay side, and the remnant of LUL was amputated completing the LUL lobectomy. A serratus muscle flap was placed between the aortic graft and pulmonary artery.

The patient was discharged home in stable condition on post-operative day 7. At 2 years of follow-up, hemoptysis had not recurred.

**Figure 3:**
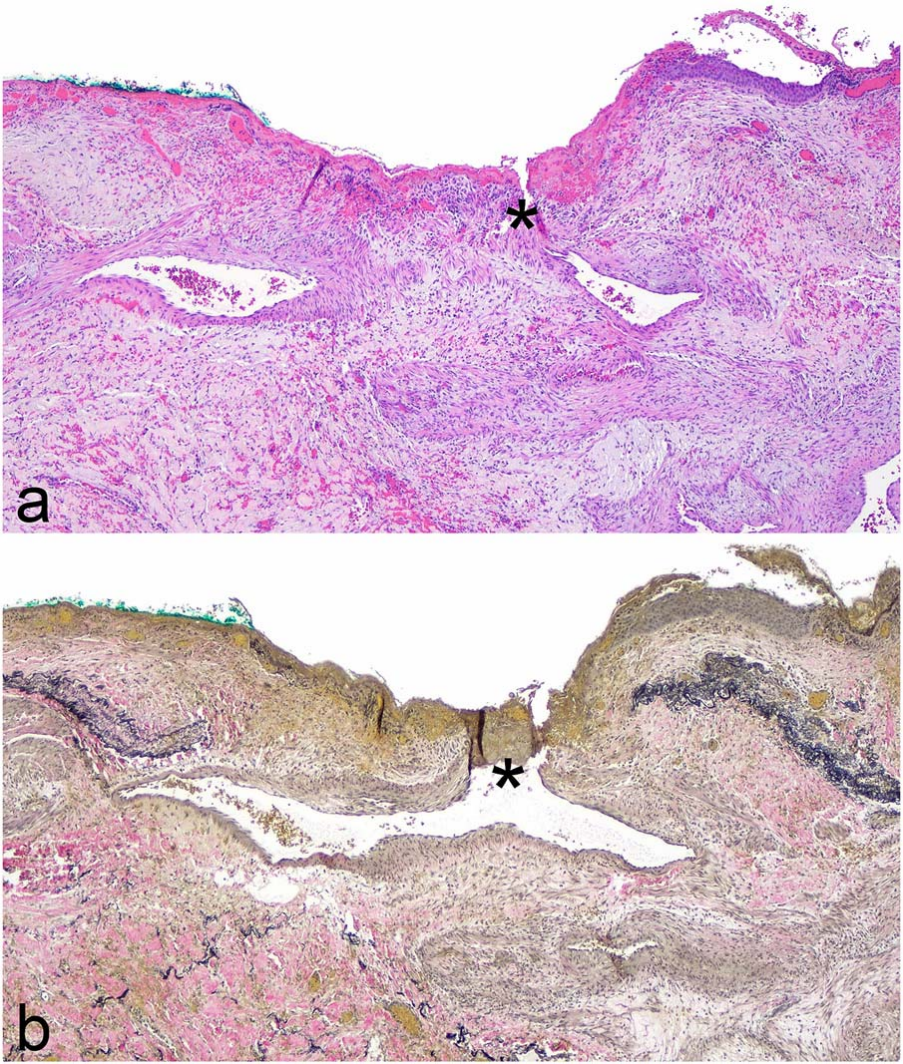
Photomicrographs of fistula between airway and small bronchial artery (*) from lobectomy specimen; (a) Hematoxylin and eosin stain, original magnification ×400; (b) elastic stain, original magnification ×400.

Pathology revealed a microscopic, partially thrombosed fistula between a small bronchial artery branch and a small airway (Fig. 3). Pathologic conclusion was that of chronic pulmonary hemorrhage secondary to bronchiolo-arterial fistula.

## Discussion

Patients with LDS are predisposed to arterial aneurysms. To our knowledge, the only published report of patients with LDS with hemoptysis is a case series of four patients [4]. The first case was a 13-year-old male with a history of aortic aneurysms who presented with massive hemoptysis and was treated with a left lower lobectomy. In the second case, a 24-year-old female presented with massive hemoptysis, which was managed conservatively. Thirdly, a 44-year-old female with a history of multiple aortic surgeries presented with hemoptysis and vascular ectasia in the left lower lobe. CT angiography (CTA) showed an aberrant collateral vessel from the LIMA, and she underwent failed coil embolization and subsequent open surgical ligation of the LIMA, which resolved her symptoms. The fourth case involved a 64-year-old male with a history of aortic aneurysms requiring multiple surgeries. He presented with massive hemoptysis, a CTA revealed recurrent aneurysms, and he passed away while receiving corticosteroid treatment. No definitive cause for the hemoptysis was identified in these four cases.

The patient presented here did not have hemoptysis until after aorta reconstruction and therefore it seems logical it was secondary to this surgery. An extra-gastrointestinal Dieulafoy lesion is possible but very rare, and the lack of arterial tortuosity histologically makes this etiology less likely. The most likely etiology is that a local inflammatory process in the LUL adjacent to the reconstructed aortic arch resulted in adhesion with neovascularization of bronchial arteries. These arteries formed a fistula to a small airway which may have been predisposed by LDS.

Bronchiolo-arterial fistulae are rare. Several case reports describe successful management of pulmonary artery-bronchus fistulae with a range of treatments including endovascular stent placement [5], coil embolization [6, 7], and pneumonectomy [8].

In summary, bronchiolo-arterial fistulae should be considered in similar cases of chronic pulmonary hemorrhage. Unfortunately, the diagnosis can be challenging to make radiologically and may only be available upon pathological review.
